# Diagnostic and prognostic relevance of circulating exosomal miR-373, miR-200a, miR-200b and miR-200c in patients with epithelial ovarian cancer

**DOI:** 10.18632/oncotarget.7850

**Published:** 2016-03-02

**Authors:** Xiaodan Meng, Volkmar Müller, Karin Milde-Langosch, Fabian Trillsch, Klaus Pantel, Heidi Schwarzenbach

**Affiliations:** ^1^ Department of Tumor Biology, University Medical Center Hamburg-Eppendorf, Hamburg, 20246, Germany; ^2^ Department of Gynecology, University Medical Center Hamburg-Eppendorf, Hamburg, 20246, Germany

**Keywords:** epithelial ovarian cancer, exosomal miRNAs, exosomes, diagnosis, prognosis

## Abstract

Exosomes are membrane vesicles that mediate intercellular communication by transporting their molecular cargo from cell to cell. We investigated whether serum levels of exosomal miR-373, miR-200a, miR-200b and miR-200c and circulating exosomes have diagnostic and prognostic relevance in a cohort of 163 epithelial ovarian cancer (EOC) patients using TaqMan MicroRNA assays and ELISA. The serum concentrations of exosomal miR-373 (*p* = 0.0001), miR-200a (*p* = 0.0001), miR-200b (*p* = 0.0001) and miR-200c (*p* = 0.028) were significantly higher in EOC patients than healthy women. The levels of miR-200a (*p* = 0.0001), miR-200b (*p* = 0.0001) and miR-200c (*p* = 0.019) could distinguish between malignant and benign ovarian tumors. While the levels of miR-373 and miR-200a were increased in all FIGO/lymph node stages (*p* = 0.0001), the levels of miR-200b and miR-200c were higher in patients with FIGO stage III–IV (*p* = 0.0001, *p* = 0.008, respectively) including lymph node metastasis (*p* = 0.0001, *p* = 0.004, respectively) than FIGO stages I–II. The increased levels of miR-200b and miR-200c were also associated with CA125 values (*p* = 0.0001, *p* = 0.0001, respectively) and a shorter overall survival (*p* = 0.007, *p* = 0.017, respectively). The levels of exosomes were excessively elevated in EOC patients (*p* = 0.0001). In all three cohorts, they were positively associated with the serum levels of exosomal miR-373 (*p* = 0.004), miR-200a (*p* = 0.0001), miR-200b (*p* = 0.0001) and miR-200c (*p* = 0.008). In conclusion, the increased levels of exosomal miR-200b and miR-200c mainly observed in advanced EOC suggest that these microRNAs may be involved in tumor progression. The high concentrations of exosomes in EOC patients imply an excessive, active exosomal secretion in EOC.

## INTRODUCTION

Exosomes, small membrane vesicles in size of 30–100 nm, are actively released from multiple cell types, including dendritic cells, lymphocytes and tumor cells by exocytosis [1]. They can mediate cell-to-cell communication by transferring proteins, lipids and nucleic acids between cells, resulting in the transformation from wild type cells into malignant cells [2, 3]. The amount of secreted exosomes has been associated with tumor invasiveness both *in vitro* and *in vivo*, and to promote migration and proliferation of tumor cells [4].

Exosomes contain microRNAs (miRNA) and their content depends on their cell origin. The process of sorting and packaging of miRNAs into exosomes is selective, favoring certain miRNAs for exosomal cargo over others [5, 6]. Apart from their active release by exosomes, other cell physiological events, such as apoptosis and necrosis, also release miRNAs into the blood circulation [7, 8], then miRNAs form complexes with specific RNA-binding proteins, e.g., AGO2 and HDL proteins [9] or are integrated in apoptotic bodies [10]. MiRNAs, a family of evolutionary conserved, small non-coding RNA molecules, inhibit gene expression post-transcriptionally by binding specifically to the 3′untranslated-region (3′UTR) of their target mRNAs, leading to the suppression of protein expression or cleavage of their target mRNAs [11]. Computational analyses indicate that one miRNA has binding affinity to hundreds of different mRNAs and hence, miRNAs are involved in the regulation of various cellular processes, such as development, differentiation, proliferation and tumor development [12]. In mammals, they are believed to downregulate approximately 50% of all protein-coding genes [13]. As miRNAs loci frequently map to fragile chromosomal regions harboring DNA amplifications, deletions or translocations, their expression is often deregulated during tumorigenesis, contributing to tumor progression and metastasis [14].

Therefore, based on their biological functions and the possibility to quantify them in real-time in patient blood, exosomal miRNAs may be a new promising class of potential non-invasive biomarkers for EOC. Its high mortality may be due to the fact that EOC is frequently late symptomatic and, therefore, detected too late. Approximately 70% of patients are diagnosed with advanced FIGO stages (III or IV) and have a 5-year survival rate of less than 40%, whereas patients who are diagnosed with FIGO stage I or II have a longer 5-year survival rate of 70−90% [15]. Current diagnostic methods for detection and monitoring of EOC mainly include pelvic examination and transvaginal ultrasound and measurement of serum biomarker CA125 (carbohydrate antigen 125) [16, 17]. However, these methods are not sufficiently specific to diagnose EOC at an early stage, since e.g., CA125 is only elevated in approximately 50% of stage I, and 70%–90% of advanced diseases [18]. Screening of exosomal miRNAs could improve therapy and may provide information on aberrant signaling pathways involved in EOC, that could be blocked by a chosen targeted therapy. For the present study, we selected miR-373 [19, 20] and the miR-200 family members [21], because these miRNAs have cancer-specific characteristics. In particular, the miR-200 family has been described to have multiple functions in many cancer types [21, 22]. Of interest was also miR-373, because our data showed an association of the serum levels of exosomal miR-373 with triple negative and more aggressive breast carcinomas [23].

## RESULTS

### Work flow

**Figure 1:**
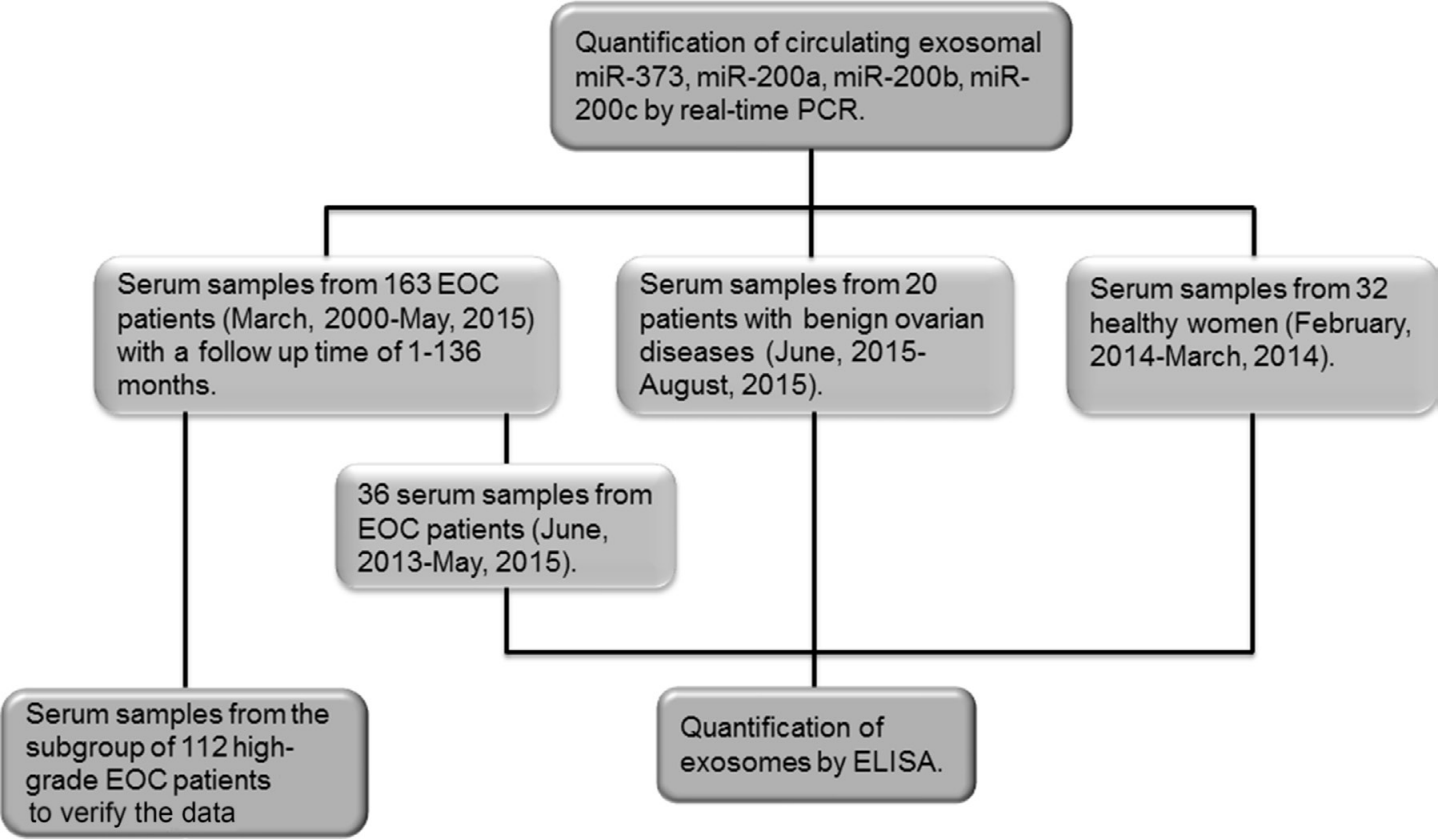
Workflow of the present study

Six miRNAs (miR-141, miR-373, miR-200a, miR-200b, miR-200c and miR-429) were quantified from exosomes derived from the serum samples of 163 EOC patients, 20 patients with benign ovarian diseases and 32 healthy women. As miR-141 and miR-429, which also belong to the miR-200 family, were not detectable in exosomes, all further analyses focused on miR-373 and the miR-200 family members: miR-200a, miR-200b and miR-200c. We also validated these exosomal miRNAs in a subgroup of 112 high-grade patients, and the cohorts of patient with benign ovarian tumors and of healthy women. Following the quantification of exosomal miRNAs, serum of 36 EOC patients, 20 patients with benign ovarian diseases and 32 healthy women were still available for the quantification of exosomes (Figure 1). We selected miR-373 and the miR-200 family members for our study, because they have been described as tumor suppressor genes and oncogenes with multiple cancer-specific functions [27, 28].

### Diagnostic relevance of exosomal miR-373, miR-200a, miR-200b and miR-200c

**Figure 2:**
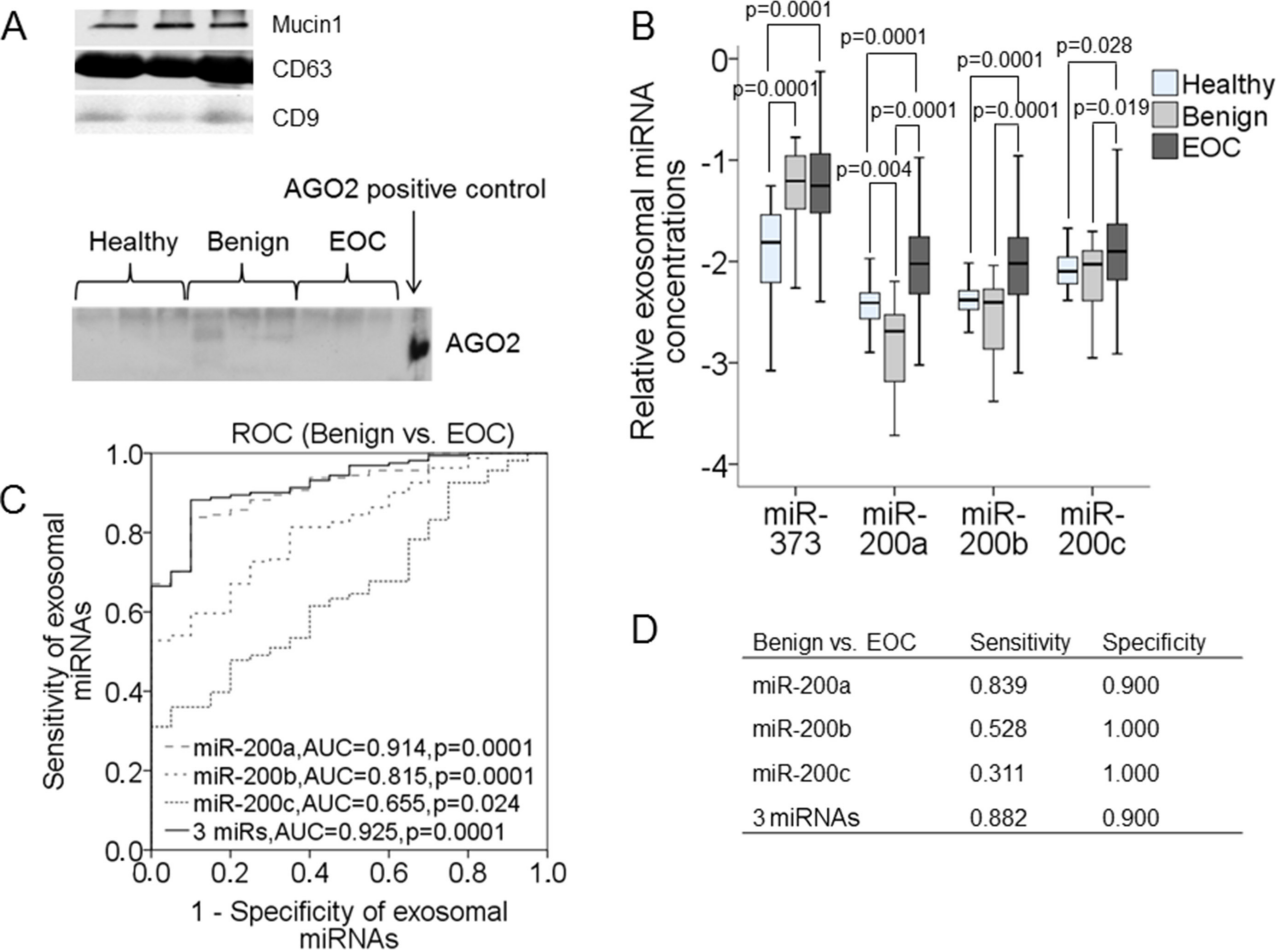
Quantification of exosomal miR-373, miR-200a, miR-200b and miR-200c in the serum of healthy women, patients with benign ovarian diseases and EOC patients Exosomes were extracted and pelleted from serum and analyzed by Western blot using antibodies specific for the exosome proteins Mucin1, CD63 and CD9. The Western blot shows a representative example of the exosome extraction of three EOC patients (**A, above**). Western Blot with non-lysed exosomes and an antibody specific for AGO2 protein. AGO2 protein is only detectable in HeLa cell protein that serves as a positive control (**A, below).** The box plot compares the exosomal miRNA concentrations in the serum of healthy women (*n* = 32), patients with benign ovarian diseases (*n* = 20) and EOC patients (*n* = 163) (**B**). ROC analyses show the profiles of sensitivity and specificity of exosomal miR-200a, miR-200b, miR-200c and their combination to distinguish benign ovarian diseases from EOC (**C**). Summarization of sensitivities and specificities of exosomal miR-373, miR-200a, miR-200b, miR-200c and their combination (**D**).

For quantification of our panel of exosomal miRNAs, we extracted at first exosomes from serum, and verified them on a Western blot using 3 different antibodies specific for the exosomal markers: Mucin1, CD63 and CD9 (Figure 2A, above). As reported by Lobb et al. [29], polymeric-based precipitation of exosomes does not allow complete depletion of miRNA-containing AGO2-complexes, therefore, to examine whether our exosome solution is contaminated by cell-free AGO2-bound miRNAs, we also carried out a Western Blot using an antibody specific for AGO2 protein. We could not detect any AGO2 protein in non-lysed exosome solutions of the three populations (healthy women, patients with benign ovarian diseases or EOC), indicating that the extracted exosomes may be quite pure (Figure 2A, below). However, this finding does not exclude that our exosome solution may contain traces of cell-free AGO2-bound miRNAs that due to the sensitivity of the Western blot were not detectable.

Then we extracted the small RNA molecules from these exosomes. Their levels were higher in both cohorts of EOC patients (*p* = 0.0001) and patients with benign ovarian diseases (*p* = 0.0001) than those in healthy women (Supplementary Figure S1). Quantification of the relative levels of exosomal miRNAs were carried out by TaqMan real-time RCR. As shown by the box plots, the serum levels of exosomal miR-373 (*p* = 0.0001,), miR-200a (*p* = 0.0001), miR-200b (*p* = 0.0001) and miR-200c (*p* = 0.028) were significantly higher in EOC patients than in healthy women (Figure 2B), whereas miR-141 and miR-429 were undetectable in exosomes (data not shown). The increase in serum levels of exosomal miR-373 was also observed in patients with benign ovarian diseases (*p* = 0.0001), whereas surprisingly, the levels of exosomal miR-200a were lower in patients with benign tumors than in healthy women (*p* = 0.004). Accordingly, the concentrations of miR-200a (*p* = 0.0001), miR-200b (*p* = 0.0001) and miR-200c (*p* = 0.019) could differ between malignant and benign tumors (Figure 2B). The significant differences in the serum levels of exosomal miRNAs between EOC patients and patients with benign ovarian diseases were reflected by the AUC values of miR-200a, miR-200b, miR-200c and their combination, which were 0.914, 0.815, 0.655 and 0.925, respectively (Figure 2C). To improve the discrimination, the concentrations of exosomal miR-200a, miR-200b, miR-200c were combined and analyzed by binary regression. This panel of exosomal miRNAs could discriminate between EOC patients and patients with benign ovarian diseases with a sensitivity of 88% and a specificity of 90%, while miR-200a alone could even differ between these both patient cohorts with a sensitivity of 84% and a specificity of 90%. Sensitivities and specificities of miR-200a, miR-200b, miR-200c and their combination (Figure 2D) were determined by the highest Youden index (sensitivity + specificity − 1).

**Figure 3:**
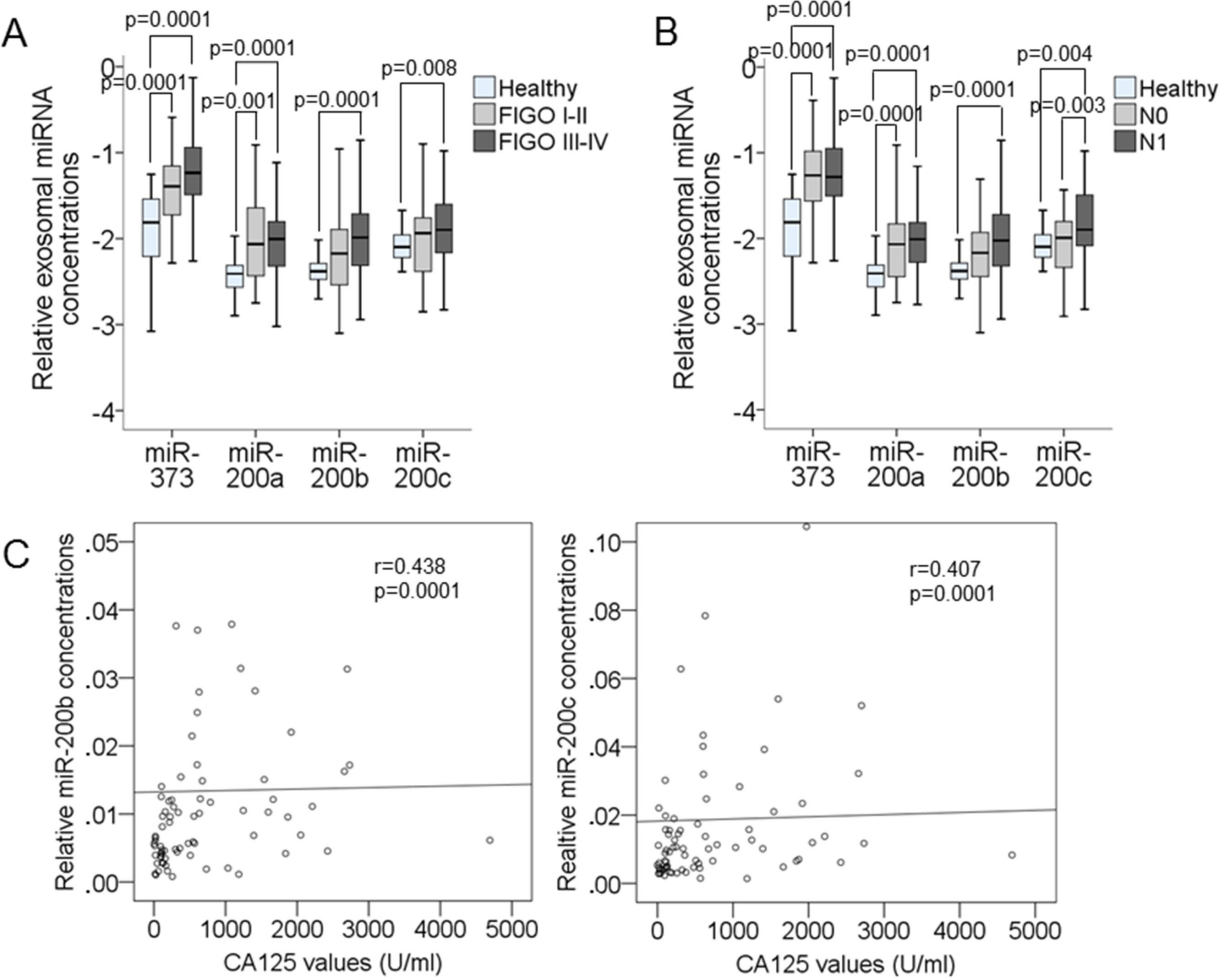
Correlations of the serum levels of exosomal miR-373, miR-200a, miR-200b and miR-200c with the clinical parameters of EOC patients The box plot compares the exosomal miRNA concentrations in the serum of healthy women (*n* = 32) EOC patients with FIGO I-II (*n* = 27) and FIGO III-IV (*n* = 118) (**A**). The box plot compares the exosomal miRNA concentrations in the serum of healthy women (*n* = 32), lymph-node negative (N0, *n* = 48) and lymph-node positive (N1, *n* = 78) EOC patients (**B**). The scatter plots show the correlations of concentrations of exosomal miR-200b and miR-200c with the CA125 values of EOC patients (*n* = 77) (**C**).

In addition, we compared the concentrations of exosomal miR-373, miR-200a, miR-200b and miR-200c with the clinical and histopathological risk factors. Supplementary Table S1 summarizes the *p* values. Compared with those of healthy women, we detected that the serum levels of exosomal miR-373 and miR-200a were increased in EOC patients with FIGO I–II (*p* = 0.0001, *p* = 0.0001, respectively) and remained increased in EOC patients with FIGO III–IV (*p* = 0.001, *p* = 0.0001, respectively). However, the serum levels of exosomal miR-200b (*p* = 0.0001) and miR-200c (*p* = 0.008) were higher in EOC patients with FIGO III–IV than healthy women (Figure 3A). These findings show that both miRNAs rather play a role in advanced cancer, and are also supported by the fact that the levels of exosomal miR-200b (*p* = 0.0001) and miR-200c (*p* = 0.004) were only elevated in lymph node-positive patients. Furthermore, the observation that the serum levels of the two other miRNAs (miR-373 and miR-200a) were increased in all FIGO stages was conform with their increase in both, lymph node-negative and -positive stages (*p* = 0.0001, Figure 3B).

These findings provoked us to investigate whether the changes in the concentrations of these miRNAs correlate with the values of tumor marker CA125. Serum CA125 assessment is currently the standard of care in performing diagnosis, following response to treatment, and predicting prognosis of EOC patients. Its sensitivity increases during cancer progression [18]. As shown by the dot plots of Figure 3C, we detected that the levels of exosomal miR-200b (*p* = 0.0001, *r* = 0.438) and miR-200c (*p* = 0.0001, *r* = 0.407) that were only upregulated in FIGO III-IV stage including lymph node-positive ovarian cancer correlated with serum CA125 values. Since no CA125 values were measured in healthy women and patients with benign ovarian diseases, we could not determine and compare its sensitivity and specificity with those of exosomal miRNAs to detect EOC.

### Exosomal miR-373, miR-200b and miR-200c exhibit potential prognostic impact

**Figure 4:**
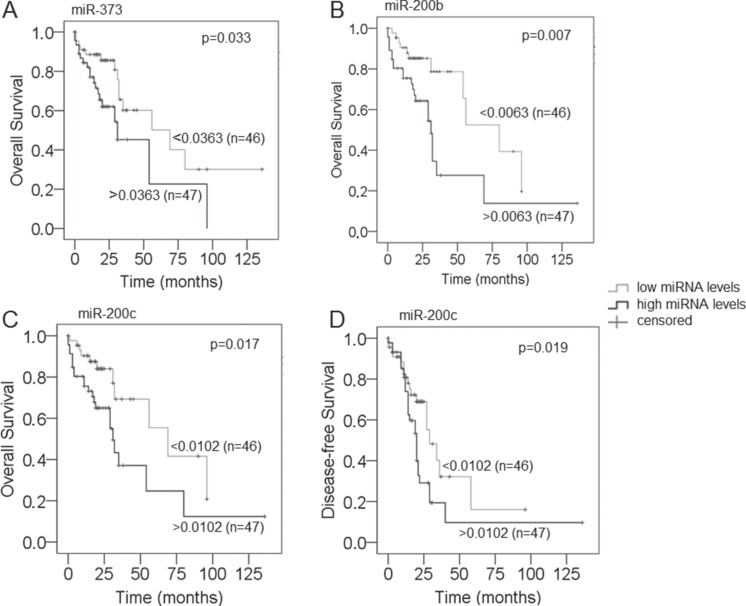
Correlations of the serum levels of exosomal miR-373, miR-200b and miR-200c with overall survival and disease-free survival of EOC patients Univariate Kaplan-Meier curves are related to low and high serum concentrations of exosomal miR-373 (**A**), miR-200b (**B**) and miR-200c (**C**) for overall survival and of miR-200c (**D**) for disease-free survival. The median values of each exosomal miRNA concentrations were used for grouping the EOC samples according to low (*n* = 46) and high (*n* = 47) transcript levels.

To assess the prognostic potential of the serum levels of exosomal miRNAs in EOC patients, Kaplan-Meier and log-rank models were carried out. The median follow-up time was 20 months (range: 1 to 136 months). As indicated in Figure 4, median values of exosomal miRNAs were used for grouping the serum samples according to low and high expression levels. The higher serum concentrations of exosomal miR-373 (*p* = 0.033, Figure 4A), miR-200b (*p* = 0.007, Figure 4B) and miR-200c (*p* = 0.017, Figure 4C) were associated with poor overall survival. In addition, the higher levels of miR-200c were associated with shorter disease-free survival (*p* = 0.019, Figure 4D). Table 1 summarizes our uni- and multivariate analyses of miRNAs with the clinical parameters of FIGO stages, grading, CA125 levels and tumor residual for overall and disease-free survival. Our data suggest exosomal miR-373, miR-200b and miR-200c to act as independent prognostic factors for overall survival, but not miR-200c for recurrence-free survival. The significant association of higher levels of miR-200b and miR-200c with poor survival supports our former data, that both miRNAs are rather involved in advanced tumors.

**Table 1:** Univariate and multivariate analyses for overall survival and disease-free survival of EOC patients

Factors	Univariate analysis		Multivariate analysis	
	HR (95% CI)	*p*-value	HR (95% CI)	*p*-value
**Overall survival**				
FIGO stage (I–II, III–IV)	13.4 (1.8–99.3)	**0.001**	7.8 (1.0–60.7)	**0.048**
Grading (G1-2, G3)	0.9 (0.4–1.8)	0.758		
CA125 (low, high)	2.3 (1.1–4.9)	**0.038**	1.4 (0.6–3.0)	0.437
Tumor residual (yes, no)	2.4 (1.1–5.4)	**0.035**	1.8 (0.8–4.4)	0.176
Exosomal miR-373	2.1 (1.0–4.3)	**0.033**	2.9 (1.3–6.8)	**0.012**
Exosomal miR-200a	1.7 (0.8–3.5)	0.220		
Exosomal miR-200b	2.7 (1.3–5.7)	**0.007**	2.8 (1.1–6.8)	**0.029**
Exosomal miR-200c	2.4 (1.2–4.9)	**0.017**	2.5 (1.1–6.1)	**0.038**
**Recurrence-free survival**				
FIGO stage (I–II, III–IV)	51.5 (3.5–769.8)	**0.004**	127.7 (0.1–1272.1)	0.953
Grading (G1-2, G3)	0.9 (0.5–1.8)	0.904		
CA125 (low, high)	2.8 (1.4–5.6)	**0.003**	1.9 (0.9–4.2)	0.102
Tumor residual (yes, no)	2.2 (1.1–4.4)	**0.024**	0.9 (0.4–2.0)	0.776
Exosomal miR-373	1.6 (0.9–2.9)	0.099		
Exosomal miR-200a	1.1 (0.6–1.9)	0.869		
Exosomal miR-200b	1.6 (0.9–2.8)	0.135		
Exosomal miR-200c	2.0 (1.1–3.6)	**0.019**	1.7 (0.8–3.6)	0.171

Significant *p* values in bold.

### Diagnostic and prognostic relevance in the subgroup of high-grade, serious EOC patients

To further analyze the above results, we further examined the exosomal miRNA levels in 112 restricted EOC patients with high grade, the most common histological subtype. We found that the results were similar (or nearly the same) to those of the whole EOC patient cohort (Supplementary Figure S2 and S3). Only, the serum concentrations of exosomal miR-373 that had prognostic value in the whole cohort of EOC patients (*p* = 0.033, Figure 4A) were not associated any more with the overall survival of high-grade EOC patients (*p* = 0.467, data not shown).

### Quantification of circulating exosomes

**Figure 5:**
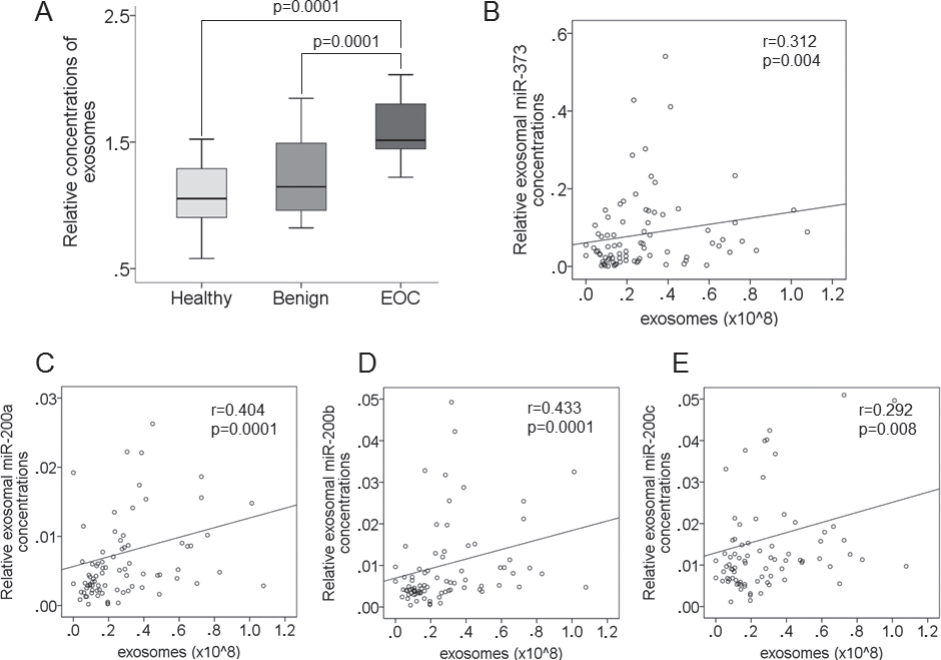
Increase in exosome levels in the serum of EOC patients and correlations between exosomes and exosomal miRNAs The box plot compares the exosome levels in the serum of healthy women (*n* = 32), patients with benign ovarian diseases (*n* = 20) and EOC patients (*n* = 36) (**A**). The scatter plots show the correlations of exosomes with the levels of exosomal miR-373 (**B**), miR-200a (**C**), miR-200b (**D**) and miR-200c (**E**) in EOC patients, patients with benign ovarian diseases and heathy women (*n* = 88).

Of the EOC patient cohort, we only had enough serum from 36 EOC patients (high-grade patients), to measure the concentrations of circulating exosomes by ELISA. We compared their exosome levels with those of 20 patients with benign ovarian diseases and 32 healthy women (Figure 5). From these three populations we extracted the exosomes, and verified them on a Western Blot using antibodies specific for the exosomal markers CD63, CD9 and Mucin1 (Supplementary Figure S5). As measured by ELISA, the exosome levels were strikingly increased in EOC patients compared with healthy women (*p* = 0.0001) and patients with benign ovarian tumors (*p* = 0.0001), indicating an excessive, active secretion of exosomes in EOC patients (Figure 5A). Bivariate correlation analyses showed that the levels of exosomal miR-373 (*r* = 0.312, *p* = 0.004, Figure 5B), miR-200a (*r* = 0.404, *p* = 0.0001, Figure 5C), miR-200b (*r* = 0.433, *p* = 0.0001, Figure 5D), miR-200c (*r* = 0.292, *p* = 0.008, Figure 5E) correlated with the secretion of exosomes. Due to the small and unbalanced patient subgroups of FIGO stages and lymph node status, it did not make sense to statistically compare the exosome levels with the clinical parameters.

## DISCUSSION

In the present study, we quantified the serum levels of exosomal miRNAs in a cohort of 163 EOC patients, 20 patients with benign ovarian diseases and 32 healthy women. Apart from our study on exosomal miR-373 in the serum of breast cancer patients [23] and the study on EpCAM-specific exosomal miR-200a, miR-200b and miR-200c in the serum of EOC by Taylor and Gercel-Taylor [30] and as far as we know, our study analyzed miR-373, miR-200a, miR-200b and miR-200c in exosomes secreted into the blood serum of cancer patients for the first time. The serum concentrations of these exosomal miRNAs could discriminate between EOC patients and healthy women, and additionally, with the exception of exosomal miR-373, between malignant and benign ovarian tumors. In particular, the increased levels of exosomal miR-200b and miR-200c associated with lymph node metastasis, FIGO stage III–IV, the tumor marker CA125 and poor overall survival demonstrate that these miRNAs may be involved in tumor progression. Besides, the levels of exosomal miR-373 and miR-200a were increased in all FIGO stages and independent of the lymph node status. These data were also confirmed by our findings derived from the subgroup of high-grade EOC patients. Moreover, we measured circulating exosomes in the serum from both patient cohorts and healthy controls, and found an excessive, active secretion of exosomes in EOC patients, compared with healthy women and patients with benign ovarian diseases.

The miR-200 family consists of miR-200a, miR-200b, miR-200c, miR-141 and miR-429. They are located in two different gene clusters: miR-200a, miR-200b and miR-429 on chromosome 1 (1p36.33), and miR-141 and miR-200c on chromosome 12 (12p13.31) [27]. If miRNAs in the same gene cluster are presumed to share a similar transcriptional expression pattern, our findings refute this argument. In our study, miR-141 and miR-429 were not detectable in exosomes, whereas exosomal miR-200b and miR-200c were mainly associated with advanced EOC and exosomal miR-200a with all ovarian tumor stages. This deviating pattern could be explained by their selective packaging into exosomes [31] or complicated processing mechanisms of primary miRNAs to mature miRNAs [32]. In this regard, Ostenfeld et al. showed that exosome-mediated secretion of miRNAs is selected during tumor progression as a mechanism to coordinate activation of the metastatic cascade [33]. During this cell-to-cell communication, tumor-derived exosomes may have the ability to propagate their oncogenic activity among cells, which possibly involves the delivery of tumor-associated miRNAs to adjacent cells [6]. Taylor and Gercel-Taylor reported that the concentration of exosomes isolated from serum samples of 50 EOC patients significantly correlated with disease progression [30]. The altered release of exosomes and discordant exosomal sequestration of miRNAs have also been found to correlate with the invasiveness of EOC cell lines [34]. These findings point to a cargo of specific miRNAs in exosomes that may represent a regulatory mechanism to maintain cancer progression. Moreover, Szajnik et al. showed that the quantification of plasma exosomes levels offers a novel approach to diagnosis and monitoring response to therapies in EOC patients [35]. The emerging role of exosomes in cancer is additionally supported by our findings showing significantly increased exosome levels in EOC patients but not in patients with benign ovarian diseases, suggesting an excessive active secretion of cancer cells.

Cancer progression is linked with the process of epithelial-to-mesenchymal transition (EMT), during which epithelial cells lose their cell polarity and cell-cell adhesion and gain migratory and invasive properties by down-regulating E-cadherin and upregulating Vimentin expression [36]. However, these changes do not fully occur in ovarian carcinoma, and are even reversed in tumor cells present in malignant peritoneal and pleural effusions [37]. The miR-200 family members seem to play a major role in the suppression of EMT and metastasis [27, 38–41]. Ectopic expression of miR-200 in cancer cell lines caused up-regulation of E-cadherin and reduced motility of cancer cells. Conversely, inhibition of miR-200 reduced E-cadherin expression, increased expression of Vimentin, and induced EMT [42]. In this regard, low-level expression of miR-200a and miR-200b in advanced ovarian tumors significantly correlated with cancer recurrence and poor overall survival, and overexpression of this miR-200 cluster inhibited ovarian cancer cell migration [42]. However, miR-200 family members seem to be versatile players [27]. Previous studies have shown that their elevated expression is a significant characteristic of EOC [27]. Elevated levels of miR-200 have been measured in EOC tissues in several studies [27], and observed in the serum of EOC patients [43, 44]. Their upregulation was associated with aggressive tumor progression and could predict prognosis and survival in EOC patients [27, 43, 44]. To date, miR-200a, 200b and 200c have only been quantified in exosomes by Taylor and Gercel-Taylor. Using microarray analysis and a small cohort of 50 EOC patients, these authors found that the levels of miR-200a, miR-200b and miR-200c derived from EpCAM-positive exosomes were higher in EOC patients with FIGO stage I, II and III than in patients with benign ovarian diseases [30]. Our data also show a cancer-specific upregulation of these miRNAs due to their ability to distinguish between malignant and benign ovarian tumors. Here, we reveal that exosomal miR-200b and miR-200c are associated with lymph node-positive status and advanced FIGO stages, while exosomal miR-200a is upregulated in all tumor stages. Elevated levels of both exosomal miRNAs, miR-200b and miR-200c correlated with the tumor marker CA125 and poor overall survival. In addition, an association between higher levels of miR-200c and poor disease-free survival was also observed.

Besides miR-200 family, we were also interested in analyzing miR-373 that has been described to promote EMT and metastasis by suppressing thioredoxin-interacting protein (TXNIP) in breast cancer [45]. Our previous studies showed a prevalence of miR-373 in the exosomal serum fraction of breast cancer patients and healthy women in comparison to the cell-free fraction [23]. Moreover, we detected that increased concentrations of miR-373 were associated with negative receptor status of breast cancer patients [23]. In another study, we found a specific influence of neoadjuvant therapy on the serum levels of miR-373 in breast cancer patients [46]. These findings provoked us to investigate miR-373 in EOC patients. Our present study shows that the expression of exosomal miR-373 was upregulated in both, malignant and benign ovarian tumors. Moreover, its levels increased in all FIGO stages, and the higher levels correlated with a shorter overall survival. In the literature, miR-373 is described as an oncogene or a tumor suppressor gene. Whereas in a study, miR-373 was reported to promote proliferation in EOC cells and to have oncogenic potential [47], in another study miR-373 was reported to have tumor suppressive characteristics. It inhibited invasion and metastasis in EOC cells by directly targeting Rab22a gene, a small GTPase [48]. Taken together, these findings show that alike to the miR-200 family, miR-373 may also have versatile functions, which among others can be explained by the fact that one single miRNA has hundreds of potential mRNA targets involved in tumor promotion or suppression.

In conclusion, our data show the diagnostic and prognostic values of exosomal miR-373, miR-200a, miR-200b and miR-200c in the serum of EOC patients who seem to have an increased active secretion of exosomes. Further analyses are needed to investigate whether packaging of these miRNAs is selective, and their role in cell-to-cell communication.

## MATERIALS AND METHODS

### Study populations

Serum samples from 163 primary EOC patients treated at the University Medical Center Hamburg-Eppendorf, Department of Gynecology, for histologically confirmed International Federation of Gynecology and Obstetrics (FIGO) stages I–IV and 20 benign ovarian tumors were included in the present study. Median ages of EOC and benign ovarian tumor patients were 60 and 46 years, and ranged from 23 to 91, and from 17 to 74 years, respectively. Serum samples of primary EOC patients were collected directly before surgery from March 2000 to May 2015. Patients were treated according to national guidelines [24]. First line treatment after primary surgery consisted of carboplatin and paclitaxel. Samples of benign ovarian tumor patients were obtained during 2015. Of the patients with benign ovarian diseases, 8 had cystomas, 3 fibromas and 2 teratomas. All patients gave written informed consent to access their blood samples and review their medical records according to our investigational review board and ethics committee guidelines. Regarding blood processing, uniform management concerning the specific described protocols was performed. The median follow-up time was 20 months (range 1–136 months). Detailed patient characteristics are summarized in Table 2. In addition, serum samples were collected during 2014 from 32 age-matched (median age 56, range 42–70) healthy women with no history of cancer and in good health based on self-report. Blood collection and experiments were performed in compliance with the Helsinki Declaration and were approved by the ethics committee (Ethik-Kommission der Ärztekammer Hamburg, Hamburg).

**Table 2:** Clinical characteristics of diagnosed EOC patients

	EOC (*n* = 163)
**Age**	
Median (range)	60 (23–91)
**FIGO stage**	
FIGO I	16 (9.8%)
FIGO II	11 (6.7%)
FIGO III	92 (56.5%)
FIGO IV	26 (16.0%)
unknown	18 (11.0%)
**Grading**	
G1, G2	37 (22.7%)
G3	112 (68.7%)
unknown	14 (8.6%)
**Lymph node metastasis**	
N0	48 (29.4%)
N1	78 (47.9%)
unknown	37 (22.7%)
**Histologic subtype**	
Serous papillary	120 (73.6%)
Other subtypes	15 (9.2%)
unknown	28 (17.2%)
**CA125**	
< 35 U/ml	8 (4.9%)
35–65 U/ml	2 (1.2%)
> 65 U/ml	67 (41.1%)
unknown	86 (52.8%)
**Postoperative residual tumor**	
Microscopie	65 (39.9%)
< 1 cm	12 (7.4%)
> 1 cm	8 (4.9%)
unknown	78 (47.8%)
**Recurrent disease at time of analysis**	
Yes	46 (28.2%)
No	48 (29.5%)
unknown	69 (42.3%)
**Survival status at time of analysis**	
Dead	34 (20.9%)
Alive	59 (36.2%)
unknown	70 (42.9%)

### Isolation of total exosomes from serum

Total exosomes were isolated by Total Exosome Isolation Reagent for Serum (Life Technologies, Austin, USA) according to the manufacturer's instructions. Briefly, 600 μl of serum, removed from cells and debris by a centrifugation step at 2,000 g for 30 min., were incubated with 120 μl of the exosome isolation reagent at 4°C, for 30 min., Following centrifugation at 10,000 g for 10 min., the total exosomes were precipitated, and exosomal proteins were extracted from the pellet with RIPA buffer (150 mM NaCl, 1% NP40, 0.5% Na-deoxycholate, 0.1% SDS, 50 mM Tris pH 8). The quality of the extracted exosomes was verified on a Western blot (as described in our previous work [23]) using 3 different antibodies specific for the exosomal markers Mucin1 (CD227, BD Biosciences, Franklin Lakes, New Jersey, USA), CD63 (AP5333b-ev, ABGENT, San Diego, California, USA) and CD9 (AP1482d-ev, ABGENT, San Diego, California, USA). The purity of the extracted exosomes (not lysed) was checked on a Western blot using the antibody specific for AGO2 protein (ab32381, abcam, San Francisco, USA). AGO2 protein isolated from HeLa cells served as positive control.

### Extraction of exosomal RNA and conversion into cDNA

Isolated exosomes were lysed by a cell disruption buffer, and exosomal RNA was extracted with the mirVana PARIS kit (Life Technologies, New York, USA) according to the manufacturer's instructions. The RNA was quantified on a NanoDrop ND-1000 Spectrophotometer (Thermo Scientific, Wilmington, Delaware, USA) and immediately reverse transcribed into cDNA. For extraction efficiency, 20 fmol of synthetic non-human cel-miR-39 was added as an exogenous spike control. Reverse transcription was performed by the TaqMan miRNA Reverse Transcription Kit (Life Technologies, New York, USA). Each reverse transcriptional reaction solution contained 0.1 μl 100 mM dNTPs, 0.66 μl MultiScribe Reverse Transcriptase (50 U/μl), 1 μl 10x Reverse Transcription Buffer, 0.13 μl RNase Inhibitor (20 U/μl), 2 μl 5 × TaqMan RT Primer, 2.77 μl nuclease-free water and 4 μl exosomal RNA solution. The reaction was carried out at 16°C for 30 min, 42°C for 30 min and 85°C for 5 min on a MJ Research PTC-200 Peltier Thermal Cycler (Global Medical Instrumentation, Ramsey, Minnesota, USA). The samples of cDNA were stored at −20°C for future usage.

### Preamplification of miR-373, miR-141, miR-200a, miR-200b, miR-200c and miR-429 cDNA

Due to the low serum levels of exosomal miR-373, miR-141, miR-200a, miR-200b, miR-200c and miR-429 in healthy women, patients with benign ovarian diseases or EOC patients, a preamplification step of cDNA was included. For an accurate normalization of these miRNAs, cDNA of the reference miR-484 was also preamplified. Four μl cDNA were preamplified in 5 μl Taq PCR Master Mix (Qiagen, Hilden, Germany), 0.5 μl 20 × TaqMan miRNA Assay mix and 0.5 μl nuclease-free water. PCR was run on a MJ Research PTC-200 Peltier Thermal Cycler (Global Medical Instrumentation): 1 cycle at 95°C for 5 min, 15 cycles at 95°C for 20 s, 60°C for 20 s and 72°C for 20 s and a terminal cycle at 72°C for 5 min.

To avoid false positive data (e.g., primer dimer formation or unspecific PCR products), the PCR products were analyzed by agarose gel electrophoresis. A negative control without any templates was included from the starting point of reverse transcription, too.

### Quantitative real-time PCR of miR-373, miR-141, miR-200a, miR-200b, miR-200c and miR-429

For quantitative real-time PCR, the miRNA-specific TaqMan miRNA assays (Life Technologies) for miR-484 (reference miRNA), miR-373, miR-141, miR-200a, miR-200b, miR-200c and miR-429 were used. In a 10 μl-reaction, 1 μl preamplified cDNA was mixed with 5 μl TaqMan Universal PCR Master Mix, 0.5 μl 20 × miRNA-specific TaqMan MicroRNA Assay Mix and 3.83 μl nuclease-free water. Quantitative real-time PCR reaction was performed at 95°C for 10 min. and in 40 cycles at 95°C for 15 s and 60°C for 60 s, on a C1000 Touch real-time PCR device (Bio-Rad, California, USA).

As there is no consensus on a reference miRNA for data normalization [25], we chose miR-484 as a reference gene to normalize our miRNA data, because this miRNA showed the smallest variation between healthy individuals, patients with benign ovarian diseases and EOC patients. For the serum samples of healthy women, patients with benign ovarian diseases and EOC patients, the mean Ct values of exosomal miR-484 were 15.12 (SD = 0.86), 13.07 (SD = 1.36) and 15.91 (SD = 1.88), respectively. Due to its stable expression, miR-484 has recently also been used as a reference gene in the serum of breast cancer patients [23, 26]. The interindividual variability of the efficiency of our procedures was controlled by spiking of cel-miR 39, too. Our measurements showed a mean Ct value of 15.27 (SD = 1.13) and a median value of 15.05, indicating that our data are relatively robust.

The obtained data of the miRNA expression levels were calculated and evaluated by the ΔCt method as follows: ΔCt = mean value Ct (reference miR-484) − mean value Ct (miRNA of interest) and the relative miRNA levels corresponded to the value of 2^(ΔCt).

### Quantification of exosomes by ELISA assay

Serum amounts of total exosomes were quantified by the Exosome Antibodies & ELISA Kit (System Biosciences, Mountain View, California, USA), which is specific for the exosomal protein CD63. At first, exosomes were precipitated from 250 μl serum, removed from cells and debris by a centrifugation step at 2,000 g for 30 min., with 63 μl ExoQuick exosome precipitation solution (BioCat, Heidelberg, Germany) and then resuspended in 200 μl exosome binding buffer according to the manufacturer's instructions. Fifty μl of these exosomal protein samples and CD63 protein standards (undiluted, diluted 1:2, 1:4, 1:8, 1:16, 1:32 and 1:64) were added to the micro-titer plate. ELISA assay was carried out following the manufacturer's instruction. The absorbance at 450 nm was measured on a spectrophotometric plate reader (Tecan, Männerdorf, Switzerland), and the amounts of CD63 protein were calculated according to the exosome protein standard curve. The quality of the extracted exosomes was verified on a Western blot using 3 different antibodies specific for the exosomal markers Mucin1 (CD227, BD Biosciences, Franklin Lakes, New Jersey, USA), CD63 (AP5333b-ev, ABGENT, San Diego, California, USA) and CD9 (AP1482d-ev, ABGENT, San Diego, California, USA).

### Statistical analyses

The statistical analyses were performed using the SPSS software package, version 22.0 (SPSS Inc. Chicago, IL). Relative expression data were log10 transformed in order to obtain normal distribution data. Statistical difference of miRNA expressions between healthy controls, patients with benign ovarian diseases and EOC patients were calculated using ANOVA with Tukey's HSD test for all pairwise comparisons that correct for experiment-wise error rate. Bivariate analyses of the Spearman-Rho test were also applied. Diagnostic power of the miRNAs was analyzed by receiver operating characteristic (ROC) curves. Areas under the curves (AUC) were calculated, assuming nonparametric distribution. Binary logistic regression was carried out to obtain the probabilities of combined exosomal miRNAs and to perform the ROC analysis. Univariate and multivariate analyses of miRNAs with the clinical parameters of FIGO stages, grading, CA125 levels and tumor residual were performed for prognostic factors using the Cox regression model. Kaplan-Meier plots were drawn on to estimate overall and recurrence-free survival, and the Log rank test was applied for statistical analyses. Missing data were handled by pairwise deletion. A *p*-value < 0.05 was considered as statistically significant. All *p*-values are two-sided.

## SUPPLEMENTARY MATERIALS FIGURES AND TABLE


