# Supplementary material for: Diagnostic and prognostic relevance of circulating exosomal miR-373, miR-200a, miR-200b and miR-200c in patients with epithelial ovarian cancer
Source: Oncotarget. 2016 Mar 2;7(13):16923–35. doi: 10.18632/oncotarget.7850 (PMC4941360; doi:10.18632/oncotarget.7850)
Supplement: Supplementary file 1 [file oncotarget-07-16923-s001.pdf]

Diagnostic and prognostic relevance of circulating exosomal miR-373, miR-200a, miR-200b and miR-200c in patients with epithelial ovarian cancer

Supplementary Materials

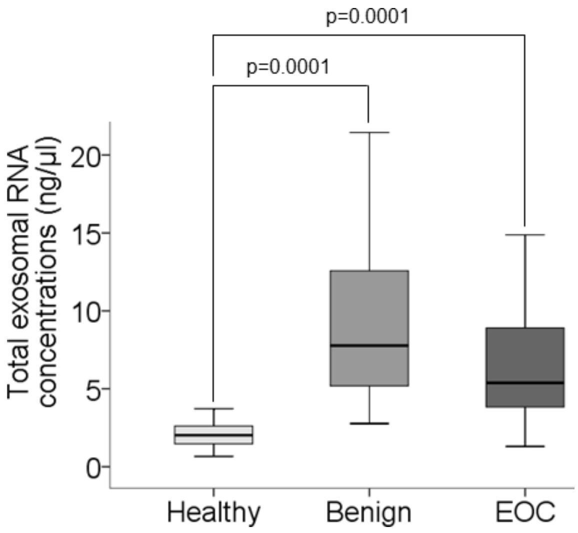

Supplementary Figure S1: Comparison of the serum levels of total exosomal RNAs in healthy women (*n* = 32), patients with benign ovarian diseases (*n* = 20) and EOC patients (*n* = 163).

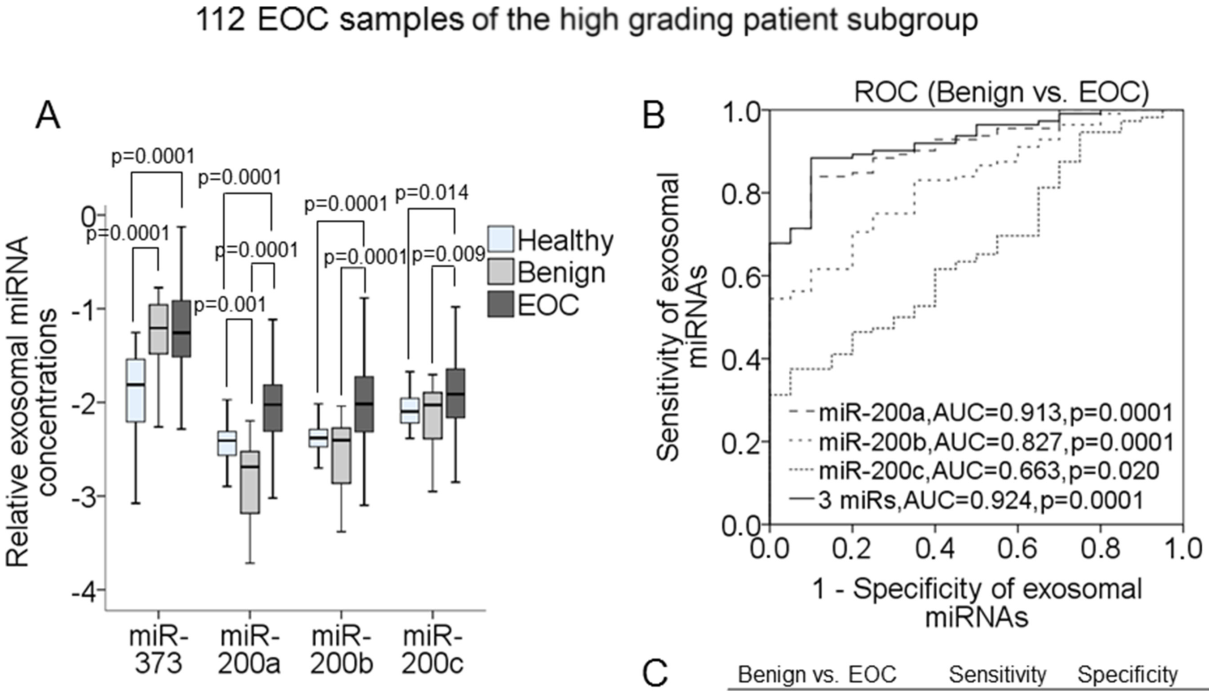

C

Benign vs. EOC	Sensitivity	Specificity
miR-200a	0.839	0.900
miR-200b	0.545	1.000
miR-200c	0.375	0.950
3 miRNAs	0.884	0.900

Supplementary Figure S2: Quantification of exosomal miR-373, miR-200a, miR-200b and miR-200c in the serum of healthy women, patients with benign ovarian diseases and high-grade EOC patients. The box plot compares the exosomal miRNA concentrations in the serum of healthy women (*n* = 32), patients with benign ovarian diseases (*n* = 20) and high-grade EOC patients (*n* = 112) (**A**). ROC analyses show the profiles of sensitivity and specificity of exosomal miR-200a, miR-200b, miR-200c and their combination to distinguish benign ovarian diseases from high-grade EOC (**B**). The table summarizes sensitivities and specificities of exosomal miR-200a, miR-200b, miR-200c and their combination (**C**).

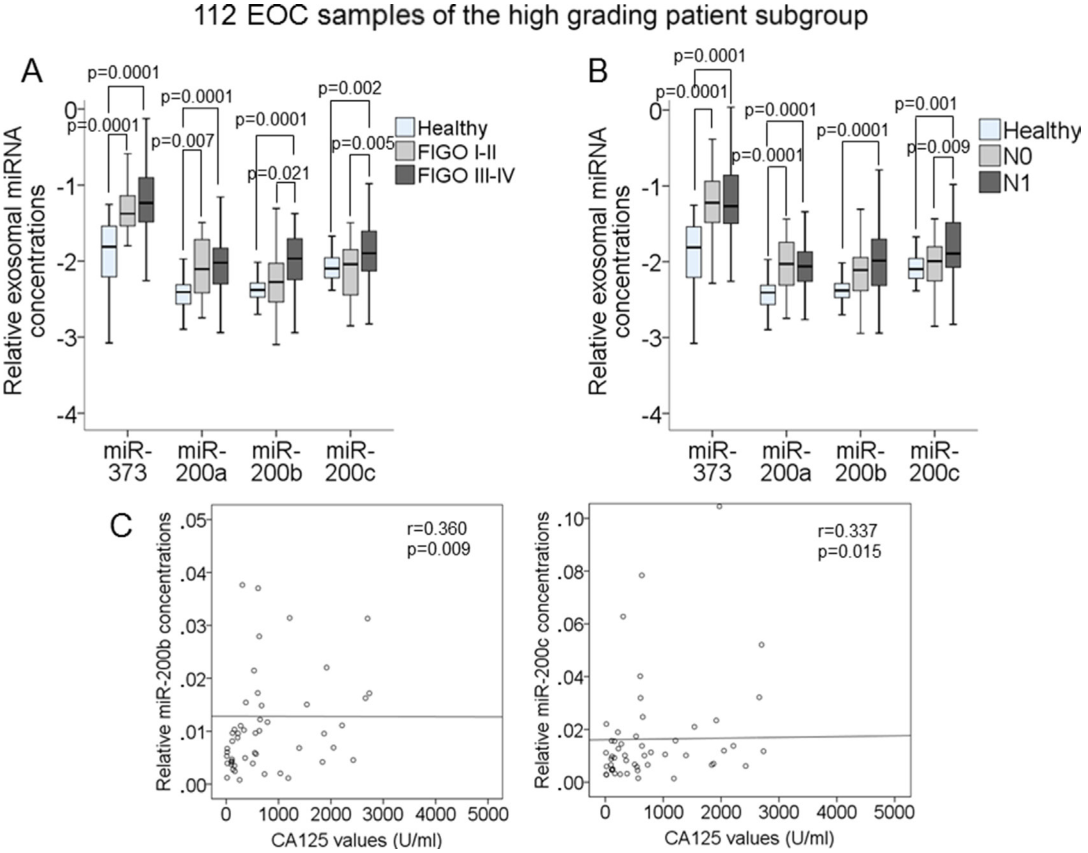

Supplementary Figure S3: Correlations of the serum levels of exosomal miR-373, miR-200a, miR-200b and miR-200c with the clinical parameters of high-grade EOC patients. The box plot compares the exosomal miRNA concentrations in the serum of healthy women (*n* = 32), high-grade EOC patients with FIGO I–II (*n* = 15) and FIGO III–IV (*n* = 90) (**A**). The box plot compares the exosomal miRNA concentrations in the serum of healthy women (*n* = 32), high-grade EOC patients with lymph-node negative status (N0, *n* = 31) and lymph-node positive status (N1, *n* = 56) (**B**). The scatter plots show the correlations of concentrations of exosomal miR-200b and miR-200c with the CA125 values of high-grade EOC patients (*n* = 52) (**C**).

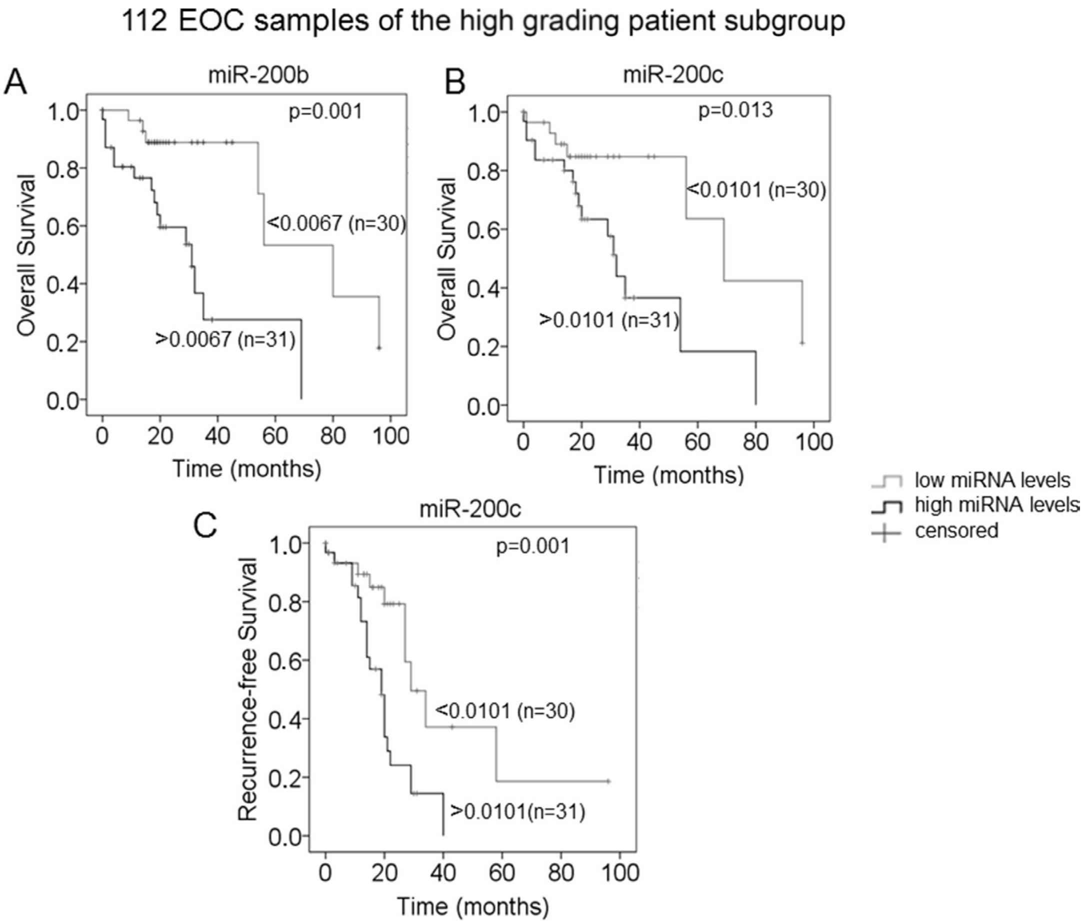

Supplementary Figure S4: Correlations of the serum levels of exosomal miR-200b and miR-200c with overall survival and disease-free survival of EOC patients. Univariate Kaplan-Meier curves are related to low and high serum concentrations of miR-200b (**A**) and miR-200c (**B**) for overall survival and of miR-200c (**C**) for disease-free survival. The median values of each exosomal miRNA concentrations were used for grouping the EOC samples with high grading according to low (n = 30) and high (n = 31) transcript levels.

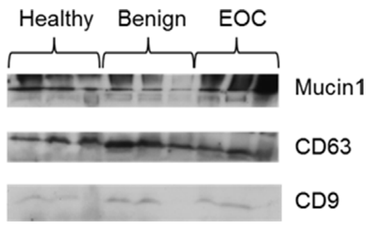

Supplementary Figure S5: Representative example of the exosome extraction by ExoQuick. Exosomes were extracted and pelleted from serum of three populations (healthy women, patients with benign ovarian diseases and patients with EOC) by ExoQuick exosome precipitation solution and analyzed by Western blot using antibodies specific for the exosome proteins Mucin1, CD63 and CD9.

Supplemenatry Table S1: Comparison of exosomal miRNA levels in the serum of EOC patients with those of patients with benign ovarian diseases and healthy women

Parameters	miR-373	miR-200a	miR-200b	miR-200c
**Total**				
Healthy vs. benign	***p* = 0.0001**	***p* = 0.004**	*p* = 0.295	*p* = 0.867
Healthy vs. EOC	***p* = 0.0001**	***p* = 0.0001**	***p* = 0.0001**	***p* = 0.028**
Benign vs. EOC	*p* = 0.863	***p* = 0.0001**	***p* = 0.0001**	***p* = 0.019**
**FIGO stage**				
Healthy vs. FIGO I–II	***p* = 0.0001**	***p* = 0.001**	*p* = 0.157	*p* = 0.865
Healthy vs. FIGO III–IV	***p* = 0.0001**	***p* = 0.0001**	***p* = 0.0001**	***p* = 0.008**
FIGO I-II vs. FIGO III–IV	*p* = 0.147	*p* = 0.993	*p* = 0.09	*p* = 0.073
**Grading**				
Healthy vs. G1, 2	***p* = 0.0001**	***p* = 0.0001**	***p* = 0.005**	*p* = 0.17
Healthy vs. G3	***p* = 0.0001**	***p* = 0.0001**	***p* = 0.0001**	***p* = 0.027**
G1, 2 vs. G3	*p* = 0.084	*p* = 0.368	*p* = 0.785	*p* = 0.894
**Lymph node metastasis**				
Healthy vs. N0	***p* = 0.0001**	***p* = 0.0001**	*p* = 0.078	*p* = 0.943
Healthy vs. N1	***p* = 0.0001**	***p* = 0.0001**	***p* = 0.0001**	***p* = 0.004**
N0 vs. N1	*p* = 0.544	*p* = 0.606	*p* = 0.059	***p* = 0.003**
**Tumor rest**				
Tumor-free vs. tumor-residual	*p* = 0.257	*p* = 0.578	*p* = 0.057	***p* = 0.041**

Significant *p* values in bold.
